# Our Collective Needs and Strengths: Urban AI/ANs and the COVID-19 Pandemic

**DOI:** 10.3389/fsoc.2021.611775

**Published:** 2021-02-10

**Authors:** Tara L. Maudrie, Kerry Hawk Lessard, Jessica Dickerson, Kevalin M. W. Aulandez, Allison Barlow, Victoria M. O’Keefe

**Affiliations:** ^1^ Johns Hopkins Center for American Indian Health, International Health Department, Johns Hopkins Bloomberg School of Public Health, Baltimore, MD, United States; ^2^ Native American Lifelines, Baltimore, MD, United States

**Keywords:** American Indian/Alaska Native, Indigenous, COVID-19, urban Indian, urban Indian health organization, coronavirus

## Abstract

The COVID-19 pandemic has raised national consciousness about health inequities that disproportionately impact American Indian/Alaska Native (AI/AN) communities, yet urban AI/AN communities continue to remain a blind spot for health leaders and policymakers. While all United States cities have been the traditional homelands of AI/AN peoples since time immemorial, urban AI/ANs are consistently excluded in local and national health assessments, including recent reports pertaining to COVID-19. Today the majority of AI/ANs (71%) live in urban areas, and many cities have strong Urban Indian Health Programs (UIHPs) that provide space for medical care, community gatherings, cultural activities, and traditional healing. Many of these UIHPs are currently scrambling to meet the needs of their AI/AN service communities during the pandemic. While the COVID-19 pandemic brought new sources of funding to UIHPs, the lack of local AI/AN data and arbitrary funding restrictions precluded some UIHPs from addressing their communities’ most immediate challenges such as food and economic insecurities. Despite these challenges, urban AI/AN communities carry the historical resilience of their ancestors as they weave strong community networks, establish contemporary traditions, and innovate to meet community needs. This article focuses on the experiences of one UIHP in Baltimore City during the COVID-19 pandemic to illustrate present-day challenges and strengths, as well as illuminate the urgency for tailored, local data-driven public health approaches to urban AI/AN health.

## Introduction

All United States cities are located on ancestral and contemporary Indigenous homelands and bear complex histories regarding American Indian/Alaska Native (AI/AN) peoples and settler colonialism (Furlan, 2017). While some cities have made strides toward acknowledging harmful colonial histories, few adequately recognize the enduring presence and needs of urban AI/ANs in their cities (Furlan, 2017). Although many AI/ANs have lived in urban areas since long before the 1950s, the relocation period of the 1950s marked an important population shift, as well as a shift in perceptions of American Indian identities (Furlan, 2017). As a result of federal relocation programs enacted in the 1950s and inadequate economic and educational opportunities in their home communities, many AI/AN families have lived in urban areas for generations (Venables, 2004; Rhoades et al., 2005). In the World War II era, approximately 40,000 AI/ANs moved to urban areas to find jobs in the then booming war industries (Weaver, 2012). In 1956, the United States federal government passed the Indian Relocation Act to encourage AI/ANs to move to urban areas with the goal of assimilating them into the general population and reducing the federal government’s trust responsibilities to Native peoples (Madigan, 1956). These trust responsibilities included honoring treaty rights to healthcare and education in perpetuity in exchange for land (Madigan, 1956). The former Commissioner of Indian Affairs claimed that the goal of relocation was harmonious with the federal Indian termination policies aimed to assimilate AI/ANs into the United States (Fixico, 2000). While this policy was effective in moving AI/ANs to urban areas, promises for housing, and employment made by the Bureau of Indian Affairs were never fulfilled (Furlan, 2017). In the words of one Bureau of Indian Affairs official, they were purposely given a “one-way ticket” from reservation to urban poverty (Furlan 2017; Nesterak 2019).

The urban relocation policies of the 1950s had harmful effects that ultimately resulted in risks to AI/AN health and well-being stemming from social determinants exacerbated by relocation (Burt, 1986).

### Poverty

When gainful employment was not realized, many AI/ANs who relocated fell into poverty and some, into homelessness (Fixico, 2000). Today, poverty is a major concern for urban AI/ANs, with approximately half of all non-elderly AI/ANs living with incomes at or below the federal poverty level (Urban Indian Health Commission, 2007).

### Food Insecurity

Although residing in an urban area is associated with greater food security for other races, national data show that urban AI/ANs are 1.5 times more likely to be food insecure than rural AI/ANs (Morton and Blanchard, 2007; Bustillos et al., 2009; Jernigan et al., 2017). As shown in Table 1, Urban Indian Health Programs (UIHPs), and their clients are ineligible for many federally supported food and health related programs offered to primarily reservation based federally recognized tribes and tribal organizations. Some programs are available for Tribal citizens to apply for, but require a local organization to house the program, which is not always possible. The United States Department of Agriculture, 2016 resource guide for AI/ANs makes no mention of urban AI/AN-specific nutrition, agriculture, or housing supports (United States Department of Agriculture, 2016).

**TABLE 1:** Examples of social support programs highlighted in the United States Department of Agriculture, 2016 Resource Guide for American Indians and Alaska Natives (2016).

	Health programs and supports	Agency	Eligibility	UIHP eligibility
*Agriculture Programs*	Local food promotion program	Agricultural marketing service	Tribes; tribal organizations	UIHPs ineligible
	Microloans (for direct farm ownership and direct operating loans)	Farm service agency	Tribal citizens; tribal organizations	UIHPs clients may be eligible to apply
				UIHPs ineligible
	Farmers market promotion program	Agricultural marketing service	Tribes; tribal organizations	UIHPs ineligible
*Food Access Programs*	The food distribution program on indian reservations	Food and nutrition service	Income-eligible tribal citizens that reside on a reservation, on designated areas near reservations, or in Oklahoma	UIHP clients ineligible
				UIHPs ineligible
	Commodity supplemental food program (CSFP)	Food and nutrition service	Tribal elders at least 60 years of age (mostly offered on reservations) and residing in one of the states or on one of the indian reservations that participate in CSFP	UIHP clients may be eligible for state specific CSFP
				UIHPs ineligible
	Senior farmers’ market nutrition program (SFMNP)	Food and nutrition service	Tribes	UIHPs ineligible
*Housing*	Housing preservation and revitalization demonstration loans and grant	Rural development	Rural areas only	UIHPs ineligible
			Tribes; tribal organizations	
	Single family housing rent assistance	Rural development	Rural areas only	UIHP clients ineligible
			Tribal citizens	UIHPs ineligible

### Access to Healthcare

Over the last century, federal legislation has guaranteed healthcare for citizens of federally recognized AI/AN tribes (Snyder Act, 1921; Urban Indian Health Commission, 2007). Yet, these rights are not fully realized by AI/ANs who move to urban areas, as most Indian Health Service full ambulatory clinics and hospitals are located on reservation lands (Urban Indian Health Commission, 2007). To meet the needs of urban AI/ANs, the Indian Health Service (IHS) provides contracts and grants to 41 Urban Indian Health programs (UIHPs) (Indian Health Service, 2018). Despite the efforts of these programs, the Urban Indian Health Institute (UIHI) had documented that lack of adequate health services are a serious problem for most urban AI/AN families (Urban Indian Health Institute, 2004). On average, UIHPs receive approximately half of their funding from IHS while the remainder of their funding is composed of federal health grants, state contracts, and foundation grants (National Council of Urban Indian Health, n.d.-a). Urban AI/ANs experience the same health issues as AI/ANs nationwide, but these problems are exacerbated due to reduced access to Native-specific resources for health, social support, and cultural activities (Urban Indian Health Commission, 2007; Norris et al., 2012; Indian Health Service, 2018).

#### COVID-19

Given the intersection of poor access to adequate healthcare, food insecurity, and poverty, urban AI/ANs were severely disadvantaged when COVID-19 hit. The Centers for Disease Control and Prevention (CDC) has reported that certain underlying medical conditions, including obesity, type 2 diabetes, and heart disease, put individuals at higher risk for severe illness from COVID-19 (Center for Disease Control and Prevention, 2020a). Nationally AI/ANs are disproportionately affected by these conditions (Cobb et al., 2014; Hutchinson and Shin, 2014; Indian Health Service, 2019). CDC data show that AI/ANs are 3.5 times more likely to contract COVID-19 and to be hospitalized than non-Hispanic Whites in the United States (Center for Disease Control and Prevention, 2020b; Center for Disease Control and Prevention, 2020c; Center for Disease Control and Prevention, 2020d; Hatcher et al., 2020). Additionally, the age adjusted mortality rate for COVID-19 was 1.8 times higher in AI/ANs than in non-Hispanic Whites (Arrazola et al., 2020). As with all races, COVID-19 mortality rates appear to be increasing with age for AI/AN peoples, but the largest mortality disparity between AI/ANs and non-Hispanic whites is in the age group of 20–49 years (Arrazola et al., 2020). COVID-19 mortality rates by both sexes for AI/ANs are 1.8 times that of non-Hispanic Whites of the respective sex (Arrazola et al., 2020). Additional considerations for AI/ANs that may increase risk for COVID-19 include; historical medical mistreatment that may make AI/AN individuals reluctant to seek care, high levels of perceived discrimination in healthcare, and that nationally nearly one-third of AI/ANs under 65 years of age do not have health insurance (Burgess et al., 2008; Findling et al., 2019; Center for Disease Control and Prevention, 2020e). When combined with high levels of poverty, multigenerational and crowded housing, and inadequate funding of UIHPs, the risk of contracting COVID-19 intensified for urban AI/ANs (Moore et al., 2020).

Additional pandemic complications, such as mass lay-offs and food and sanitary supply shortages, exacerbated conditions. In the early stages of the pandemic, many people (especially people of color) lost their jobs and were left without the financial means to afford housing bills (Kantamneni, 2020; Watson et al., 2020). While many UIHPs received COVID-19 federal funding, restrictions on how the funding could be spent (i.e., restrictions on direct support for food, housing or utility assistance) precluded UIHPs from meeting community members’ most immediate needs for food, rent and utility assistance. The disconnect between the policies that created these restrictions and the community members’ survival needs signify the importance of critically examining whose interests these restrictions serve: the federal government’s or AI/AN peoples?

## Perspectives From an East Coast Urban Native Community

The remainder of this article focuses on one east coast urban Native community’s experiences and innovations during the COVID-19 pandemic as a case example to inform improved public health response to urban AI/AN needs and human rights. Despite failed government policies and inadequate health and social services, Native peoples have found each other in cities and built strong communities—often with added identity beyond tribal citizenship which includes proudly adding “urban Indian” to one’s tribal identity (Fixico, 2000). Since time immemorial, Baltimore City and the surrounding areas have been the ancestral lands of the Piscataway and Susquehannock peoples (Maryland State Archives, n.d.). Due to relocation policies and growing urbanization, Baltimore City and the six surrounding counties are now home to a strong community of over 25,000 urban AI/ANs, representing many tribal affiliations, with a notable presence from the Lumbee Tribe of North Carolina (Urban Indian Health Institute, 2018; Minner, 2019). Native American LifeLines (NAL) is a leading urban AI/AN health service center, established in 2000 as a 501c3 non-profit organization and Title V IHS contracted UIHP that serves Baltimore and surrounding counties (Native American LifeLines, n.d.-a). NAL employs seven staff representing six tribes and two allies who provide behavioral health and substance use counseling, medical case management and referrals, dental care, health promotion, and cultural programming to AI/AN community members (Native American Lifelines, n.d.-b).

### Needs Assessment: Health and Social Concerns

In the first few months of the pandemic, NAL staff contacted over 700 AI/AN clients in two rounds of phone calls to provide health education regarding COVID-19, assess community members’ needs during the pandemic, and determine what health information resources community members found to be acceptable. Several main themes emerged from this outreach including needs for nutritious foods, hygiene and cleaning supplies, financial assistance with rent and utilities, and need for reliable access to health information.

NAL staff created innovative plans to meet community members’ needs for food, cultural activities, and health information. NAL applied for emergency COVID-19 funding from the CDC (distributed through the National Council of Urban Indian Health) with the goal of creating holistic wellness boxes to mail to homes or distribute through pick up locations in Baltimore City, as well as to provide rent and utility assistance to community members in need. These boxes were to include traditional, cultural, and prepackaged foods, as well as fresh produce, cultural craft kits, hand sanitizer, face masks and COVID-19 educational materials specific to AI/ANs. They also made plans to assist with financial shortfalls for rent and utilities. However, when funds were awarded, restrictions were announced that altered these plans. Funding could not be spent on food, any property that would outlive the funding cycle, or direct housing/rent assistance (Hawk Lessard, email, September 24, 2020).

The inability of NAL to provide foods as the first line of COVID-19 support is particularly concerning. Without access to the Food Distribution Program on Indian Reservations (FDPIR) or the Commodity Supplemental Food Program, urban AI/ANs have limited emergency food relief options (United States Department of Agriculture, 2016). Many AI/AN Baltimoreans live in disadvantaged areas where primarily nutrient poor, calorically dense foods are accessible at local convenience stores. Many do not own vehicles or have funds to utilize private ride sharing options to travel to grocery stores outside of their neighborhoods. Thus, during the pandemic, Baltimore AI/ANs are faced with a lesser-of-two-evils dilemma: to access poor quality foods through corner or dollar stores in their immediate neighborhoods or risk contracting COVID-19 by using public transportation to travel to grocery stores.

Despite funding restrictions, NAL exercised creativity and determination to meet community needs. NAL partnered with another community organization to distribute fresh produce to community members most in need of food. They were able to use the CDC funding distributed through the National Council of Urban Indian Health to successfully produce wellness boxes that incorporated hand soaps, handmade cloth masks, hand sanitizer, health information, cultural activities, traditional recipes and nutrition education materials. Since NAL was unable to directly provide foods in their wellness boxes, they provided nutrition education handouts, can openers, and strainers along with education (e.g., rinse canned fruits and vegetables to reduce excess sodium and sugar). NAL is also offering community events and talking circles via video conference and is helping to improve broadband access for community members by dedicating office space for community members to access internet. NAL is also partnering with local churches to arrange future “drive-through” and walk-up distributions for school supplies, flu vaccinations, and other wellness materials.

### FUNDING CHALLENGES

The Indian Health Service (IHS) was founded in the 1950s to provide healthcare for Native peoples, but it was not until 1976 that their budget included urban AI/AN health services (Rhoades et al., 2005). Today, a single line item totaling less than 1% of the IHS budget is available to UIHPs (Joseph et al., 2017). The smallest percentage of funding is directed to serve the largest number of Native peoples (those living in urban areas)—a funding injustice that perpetuates health inequities and violates treaty rights of tribal citizens.

The Indian Health Service designates UIHPs as full ambulatory, limited ambulatory, or outreach and referral services, classifications which relate to funding for programs and scope of community health work (Tuomi, 2017). NAL is classified as an outreach and referral service and as such they do not employ their own physicians, nurses, or provide direct healthcare to community members, except for direct behavioral health and dental services. Given limited staff and funding, there are several issues that reflect the lack of capacity that small UIHPs, like NAL hold. For example, NAL rents their office and as such does not have a commercial kitchen, commercial refrigerators or have adequate space for cultural activities, like sweat lodges or medicine gardens. Several organizations offered to donate food to NAL, but without a way to store or quickly distribute food, they were unable to accept these donations.

While urban AI/AN health is extremely underfunded, the pandemic brought a rush of new funding to UIHPs from a variety of sources—including the CARES act and the Families First Coronavirus Response Act. These new funding sources, while much needed, were overwhelming to small UIHPs that are now tasked with even greater administrative burdens (Hawk Lessard, personal conversation, September 3, 2020). The new rush of funding was crisis-oriented; it could be used for sanitary supplies, health education materials, and COVID-19 testing but could not be used to fund permanent staff positions, buy property (including delivery vehicles or commercial refrigeration), or serve community members’ priority needs (i.e., food, utility, and rent assistance).

While funding restrictions were a temporary set-back, NAL redirected their focus to how the funding could be used to improve community health. NAL plans to purchase at home diagnostic COVID-19 tests for community members and have been able to reimburse for purchased and referred care due to COVID-19 concerns (including COVID-19 tests administered outside of NAL). Although NAL was unable to use funds to create permanent staff positions, they temporarily contracted community health workers to create and distribute wellness boxes. For community members who lost employment and therefore health insurance, NAL was able to cover co-pays, medical care, and prescription costs. NAL continues to provide health education through social media, the community’s preferred medium for health education.

### Invisibility of Urban AI/ANs Living in the Baltimore Area

Mainstream data collection practices by federal, state, and local health departments frequently misclassify or completely omit AI/AN data, resulting in significant undercounting of AI/ANs and their attendant needs (Urban Indian Health Institute, 2020). Racial misclassification contributes to the “invisibility” of urban Indians and hinders UIHPs’ ability to respond to and reduce health inequities, including those exacerbated by the COVID-19 pandemic. Further, fighting against racial misclassification adds to UIHPs’ already full workloads.

Since 2015, NAL has advocated for disaggregating Native data to uncover urgent issues for their community including HIV/AIDs infection and testing rates, and opioid related overdose and fatality data. Despite their persistent activism, the Baltimore AI/AN community’s concerns were not initially reflected in reporting mechanisms for COVID-19. For the first months of the pandemic, Baltimore City COVID-19 data was completely unavailable by race and was later classified as Black, White, Asian or Other (Miller, 2020; Richman, 2020). NAL staff contacted the Baltimore City Health Department to ask for disaggregation of AI/AN data lumped into the “Other” category and received no response.

Amid the pandemic, protests against police brutality, and calls for racial justice, a prominent Christopher Columbus statue was thrown into Baltimore’s Inner Harbor. The sudden media attention around this action drew the attention of the district’s City Councilman, who had a prior relationship with NAL. This councilman met with a local Indigenous activist group (including NAL staff) to ask how he could support the local AI/AN community during the pandemic. The Indigenous activist group cited lack of health data as a critical issue for Baltimore AI/AN. As a result of this meeting, the Councilman wrote a letter to the Baltimore City Health Department requesting they update COVID-19 racial classification categories to include AI/AN. Since then, the City has started to “report” AI/AN data, although it is unclear if the Baltimore City Health Department is now reporting the data for AI/AN peoples from the beginning of the pandemic or if the current available AI/AN only represents testing and cases since updating the racial categories for COVID-19 data. This lack of clarity is concerning as NAL is unable to discern if there has been a spike in AI/AN testing and cases, or if delayed testing numbers are just now being reported. Nonetheless, in the earliest stages of the pandemic, NAL was left without the proper data to document the rates of COVID-19 testing and infection of their service population and was forced to rely on anecdotal evidence to justify needs to funders and policymakers. Accurate, real-time data are vital for UIHPs to respond to local needs with programs and interventions that match community priorities.

## Discussion and Recommendations

NAL has faced challenges during the pandemic, but successfully navigated partnerships with other community organizations—including a Black-led food sovereignty movement within Baltimore City—to serve the AI/AN community. In this paper, we discussed three main issues spotlighted by COVID-19: restrictions around funding, meeting the immediate needs of the service community, and inadequate data due largely to racial misclassification.

The inadequate funding of UIHPs is a violation of the federal government’s trust responsibility to AI/AN peoples that perpetuates health inequities (Crevier, 2020). There is a need to examine the funding structures of UIHPs and how they impact the health and well-being of urban AI/ANs, especially during the current pandemic. The challenges urban AI/ANs face deserve specific and immediate attention from Indian Country’s leaders and policymakers.

While small UIHPs are advocating at the local level, there are also national advocacy organizations, such as the National Council of Urban Indian Health (National Council of Urban Indian Health, n.d.-b), who works to support and develop healthcare programs for urban AI/ANs. Further, the Urban Indian Health Institute (UIHI) is the only tribal epidemiology center that specifically represents urban AI/ANs; they work to build capacity and promote urban AI/AN health (Urban Indian Health Institute, n.d.). These organizations provide support to urban AI/AN communities and would benefit greatly from additional resources.

Regarding racial misclassification, federal, local, and state health departments should move beyond simple consultation with local UIHPs and instead form meaningful collaborations to provide data and resources to UIHPs. This will enhance UIHPs’ ability to serve their urban communities. It is also imperative that mainstream health departments and entities recognize that their views of health equity are built on Western epistemologies (Echo-Hawk, 2019). Indigenous peoples remain particularly invisible in urban landscapes and the unique intergenerational effects of previous pandemics, forced relocation, historical trauma, and cultural genocide that they have experienced are largely ignored (Brave Heart and DeBruyn, 1998; Duran et al., 1998; Brave Heart et al., 2011). One of the most essential ways mainstream health entities can support UIHPs to achieve Indigenous health equity is by critically evaluating and revising their data collection practices in true partnership with local UIHPs. The UIHI offers concrete recommendations for revising data collection measures and analyzing data in a way that honors tribal data sovereignty (Urban Indian Health Institute, 2020). An important initial step includes creating and enforcing mandates for collection of race and ethnicity in health data that utilize local, state, and federal funds (Urban Indian Health Institute, 2020). Data collection and analyses should consider allowing for multi-racial identities, as AI/AN peoples are one of the largest growing multi-racial groups in the United States (Norris et al., 2012). This can be achieved by defining AI/AN populations as inclusively as possible during analyses by using AI/AN in combination with one or more races (Urban Indian Health Institute, 2020). Weighted sampling and oversampling of AI/AN populations should be utilized to ensure that “invisible” populations are still counted, and their data is available (Urban Indian Health Institute, 2020). When reporting data, it is recommended to avoid using “multi-racial” or “other” as categories, instead consider breaking into AI/AN alone, and AI/AN in combination with one or more races (Urban Indian Health Institute, 2020). Using categories like “other” or “multi-racial” rarely yield meaningful results, for example, Baltimore City COVID-19 data was originally reported as African American, White, Asian, and Other (Miller, 2020). This sort of categorization led to confusion about whether or not AI/AN data was being collected at all, if AI/AN data was lumped into the other category, or what happened to the data of those who identified as multi-racial. Lastly, reporting strengths-based and positive outcomes helps to highlight the many successful health initiatives and innovations of AI/AN communities (Urban Indian Health Institute, 2020). While these general recommendations serve as a starting point, they do not replace direct and meaningful collaboration with local UIHPs. Each urban AI/AN community holds unique health challenges and strengths, and data priorities vary greatly from community to community.

Urban AI/AN individuals, families, and communities find themselves in cities and regions where they came for desperately needed job opportunities or have lived for many generations, in some cases due to federal policies of relocation. Despite the challenges urban AI/ANs have faced, their narrative is one of survivance and resilience driven by their reclamation of space, and creation of communities and traditions that reflect the collective strengths of the many tribal nations represented in their cities. Though they may be near or far from their traditional homelands, they share the strengths of their relatives and ancestors while forging a new collective urban Native identity. We need to honor the strengths of urban AI/AN communities and care for their health and well-being through prioritizing funding, appropriate data collection and analysis, and ensuring urban AI/AN needs are met during the COVID-19 pandemic and beyond.

## Data Availability

The original contributions presented in the study are included in the article/Supplementary Material, further inquiries can be directed to the corresponding author.
